# Quad-shot-immunotherapy: quad-shot radiotherapy with pembrolizumab for advanced/recurrent head and neck cancer

**DOI:** 10.2217/fon-2022-1146

**Published:** 2023-05-18

**Authors:** Ryan T Hughes, Rediet R Gebeyehu, John Mason Kalada, Thomas W Lycan, Bart A Frizzell, Rebecca D Kinney, Ralph B D'Agostino, Paul M Bunch, Pierre Triozzi, Wei Zhang, Cristina M Furdui, Mercedes Porosnicu

**Affiliations:** 1 Department of Radiation Oncology, Wake Forest University School of Medicine, Winston-Salem, NC 27101, USA; 2 Department of Internal Medicine, Section of Hematology & Oncology, Wake Forest University School of Medicine, Winston-Salem, NC 27101, USA; 3 Department of Internal Medicine, Wake Forest University School of Medicine, Winston-Salem, NC 27101, USA; 4 Department of Biostatistics & Data Science, Wake Forest University School of Medicine, Winston-Salem, NC 27101, USA; 5 Department of Radiology, Wake Forest University School of Medicine, Winston-Salem, NC 27101, USA; 6 Department of Cancer Biology, Wake Forest University School of Medicine, Winston-Salem, NC 27101, USA; 7 Department of Internal Medicine, Section of Molecular Medicine, Wake Forest University School of Medicine, Winston-Salem, NC 27101, USA

**Keywords:** head and neck cancer, immuno-oncology, immunotherapy, pembrolizumab, quad-shot, radiotherapy, squamous-cell carcinoma

## Abstract

Effective treatments for advanced/recurrent head and neck squamous-cell carcinoma are limited. For cases not curable by conventional local therapies, the immune checkpoint inhibitor pembrolizumab shows modest response rates. Quad-shot, a hypofractionated palliative radiotherapy regimen (14.8 Gy in four twice-daily fractions), can provide symptomatic relief, contributes to local control and may potentiate the effects of immune checkpoint inhibitors. In this study, 15 patients with advanced/recurrent head and neck squamous-cell carcinoma will be treated with pembrolizumab combined with up to three administrations of quad-shot before cycles four, eight and 13. Outcomes include disease response, survival and treatment toxicity. Correlative multiomics analysis of blood and saliva will identify molecular biomarkers of response to immune checkpoint inhibitor and the immune-related impact of quad-shot.

**Clinical trial registration:** This study (WFBCCC 60320) is registered on NCT04454489 (ClinicalTrials.gov)

## Plain language summary

Advanced and recurrent head and neck cancers are difficult to treat. Most patients receive systemic therapies, such as chemotherapy or immunotherapy, with modest rates of cancer control. We aim to test the effectiveness of an immunotherapy drug called pembrolizumab in combination with a type of low-dose radiation therapy called quad-shot. Patients will receive pembrolizumab every 3 weeks and will be treated with one to three low-dose radiation therapy courses targeted at their cancer in the head and neck approximately every 12 weeks. We plan to measure how well the cancer responds to treatment, how long this response lasts, how long patients survive and treatment side effects.

## Tweetable abstract

In a pilot study, 15 advanced/recurrent head and neck squamous-cell carcinoma patients will be treated with pembrolizumab plus quad-shot hypofractionated palliative radiotherapy. Response, survival and toxicity will be measured.

Head and neck squamous-cell carcinoma (HNSCC) is a significant cause of morbidity and mortality worldwide. Approximately 10% of patients with HNSCC have metastatic disease at presentation, 20–30% will develop metastatic disease after definitive treatment and 40–60% will progress within 3 years after curative therapy [1–8]. Immunotherapy with immune checkpoint inhibitors (ICI) has had a significant impact on the treatment of patients with HNSCC. Even patients with advanced, recurrent or metastatic (A/R/M) cancer have a chance of achieving durable cancer remission. The recommended first-line palliative treatment for the 85% of HNSCC patients with a PD-L1 level of at least 1 is single-agent immunotherapy with the PD-1-targeted ICI pembrolizumab [9]. Despite improvements in survival comparable to chemotherapy, the percentage of patients responding to single-agent immunotherapy remains below 20% (Table 1) [10–15]. Strategies to improve response rates to ICIs in patients with A/R/M HNSCC are urgently needed.

**Table 1.** Overall response rates to immunotherapy in recurrent or metastatic head and neck squamous-cell carcinoma.

Study	Agent	Overall response rate, %
KEYNOTE-012	Pembrolizumab	18
KEYNOTE-040	Pembrolizumab	15
KEYNOTE-048	Pembrolizumab	17
	Pembrolizumab + chemotherapy	36
CheckMate 141	Nivolumab	13

This study protocol aims to address this unmet clinical need by evaluating possible increased ICI effectiveness with the addition of a prospective immunosensitizing strategy and generating molecular and cellular immune-responsive models to define biomarkers predictive of treatment response and improve patient selection for ICIs in A/R/M HNSCC.

## Rationale

### Systemic therapy for A/R/M head & neck cancer

The currently preferred first-line treatment for recurrent, unresectable or metastatic HNSCC is pembrolizumab as monotherapy or in combination with platinum and 5-fluorouracil chemotherapy, per the results of KEYNOTE-048 [15], in which pembrolizumab alone improved overall survival (OS) by 4.2, 2 and 0.9 months in the PD-L1 combined positive score (CPS) ≥20 group, CPS ≥1 group and total population, respectively, compared with the control arm (treatment with cetuximab chemotherapy) [16]. Pembrolizumab combined with chemotherapy (replacing cetuximab in the previously standard combination) improved OS by 3.7, 3.2 and 2.3 months, respectively, in the corresponding CPS groups [15]. No direct comparison was planned between pembrolizumab monotherapy and pembrolizumab combined with chemotherapy, but results were comparable for patients with CPS ≥20 and ≥1. This finding led to the recommendation of pembrolizumab monotherapy as first-line treatment in patients with recurrent unresectable and/or metastatic HNSCC with CPS ≥1 and the recommendation of pembrolizumab combined with platinum and 5-fluorouracil-based chemotherapy for patients with negative PD-L1 measured by CPS. Neither pembrolizumab alone nor pembrolizumab with chemotherapy improved progression-free survival (PFS) in any of the groups compared with standard of care in KEYNOTE-048. However, when evaluating overall response rates, pembrolizumab combined with chemotherapy presented similar results as cetuximab combined with chemotherapy, whereas pembrolizumab monotherapy performed significantly worse. Overall response rates for pembrolizumab alone versus chemotherapy were as follows: 23 versus 36% in CPS ≥20, 19 versus 35% in CPS ≥1 and 17 versus 36% in the total population [16]. These findings indicate a need for methods to improve response rates after pembrolizumab monotherapy.

Combining pembrolizumab with chemotherapy may not be the ideal strategy for increasing pembrolizumab response. Although the KEYNOTE-048 study did not aim to directly compare the two arms, there are a few observations worth considering. First, the addition of chemotherapy as utilized in KEYNOTE-048 likely contributed to the observed increases in toxicity compared with pembrolizumab alone. Second, the median duration-of-response for patients responding to pembrolizumab monotherapy was high compared with the control cetuximab chemotherapy arm (22.6 vs 4.2 months in CPS ≥20, 23.4 vs 4.5 months in CPS ≥1 and 22.6 vs 4.5 months in the total population). Finally, the median duration-of-response for patients who responded to pembrolizumab combined with chemotherapy was much lower (7.1 vs 4.2 months in CPS ≥20 and 6.7 vs 4.3 months for both the CPS ≥1 group and the total population), suggesting the absence of a significant synergistic interaction or immunosensitizing effect of the chemotherapy regimen. These findings highlight the limitations of current standard of care immunotherapy-based regimens for first-line treatment of A/R/M HNSCC and the need to investigate combinations of immunotherapy-based systemic regimens with other potentially immunosensitizing therapies. A review of actively recruiting clinical studies investigating ICIs in combination with different immunosensitizing therapies is summarized in Table 2.

**Table 2.** Actively recruiting clinical studies with PD-1/PD-L1 inhibitors combined with immunosensitizing therapies in patients with advanced, recurrent or metastatic head and neck squamous-cell carcinoma.

Immunotherapy	Study phase	Immunosensitizing therapy	Therapy type	Special population	Sample size	Trial identifier
Nivolumab	Pilot	Proton SBRT	RT		19	NCT03539198
Durvalumab	I/II	Tremelimumab + SBRT	CTLA-4 inhibitor + RT		45	NCT03283605
Pembrolizumab	II	PDS0101	HPV-specific T-cell therapy	HPV+ PD-L1+	95	NCT04260126
Atezolizumab^†^	II/III	Bevacizumab	VEGF inhibitor		430	NCT05063552
Avelumab	II	N-803 + PD-L1 CAR-NK	IL-15 agonist + CAR-NK cell therapy		55	NCT04847466
Pembrolizumab	II	Ipatasertib	AKT inhibitor		52	NCT05172258
Retifanlimab	II	INCAGN01876	Anti-GITR monoclonal antibody		47	NCT05359692
Nivolumab	I	Cabozantinib	Multikinase inhibitor	HIV+	18	NCT04514484
Pembrolizumab	II	NKTR-214	IL-2 agonist		5	NCT04936841
Pembrolizumab	I	Acetylsalicylic acid Clopidogrel	Antiplatelet therapy		20	NCT03245489
Atezolizumab	I	Hypofractionated adapted RT	RT		18	NCT04477759
Pembrolizumab	II	Carboplatin and paclitaxel	Chemotherapy		35	NCT04858269
Nivolumab	II	Cabozantinib	Multikinase inhibitor		150	NCT05136196
Nivolumab	II	Ipilimumab, relatlimab	CTLA-4 inhibitor, LAG-3 inhibitor		40	NCT04326257
Pembrolizumab	I/II	CyPep-1	Intratumoral oncolytic peptide		90	NCT05383170
Pembrolizumab Nivolumab	I/II	CIMAvax	EGF vaccine		193	NCT02955290
PD-1 inhibitor	I	PT199	Anti-CD73 monoclonal antibody		41	NCT05431270
Pembrolizumab	II	Hypofractionated RT + NBTXR3	RT + hafnium oxide-containing nanoparticles		60	NCT04862455
Pembrolizumab	I/II	SQZ-eAPC-HPV	PBMC-based vaccine	HPV+	60	NCT05357898
Pembrolizumab	Pilot	Diffusing α-radiation emitter therapy	RT delivered by intratumoral radium-224 seed insertion		48	NCT05047094
Pembrolizumab	II	LN-145 + IL-2	Autologous tumor-infiltrating lymphocytes		178	NCT03645928
Ezabenlimab	I	Vesicular stomatitis virus glycoprotein (BI 1831169)	Oncolytic virus		117	NCT05155332

† The atezolizumab and bevacizumab arm will be compared in the phase II study part with the docetaxel, cisplatin or carboplatin and bevacizumab arm and the docetaxel, cisplatin or carboplatin and cetuximab arm.

CAR-NK: Chimeric antigen receptor–natural killer; PBMC: Peripheral blood mononuclear cell; RT: Radiotherapy; SBRT: Stereotactic body radiotherapy.

### Quad-shot palliative radiotherapy in head & neck cancer

Palliative radiotherapy (RT) is a mainstay in the standard of care treatment of recurrent or metastatic HNSCC [17]. Palliative RT is primarily delivered using external beam techniques over short courses using larger doses (≥3 Gy) per fraction than conventionally fractionated RT (1.8–2 Gy per fraction) in an attempt to effect local tumor regression and to relieve malignancy-related morbidity while limiting acute toxicity. Various fractionations have been reported, with the quad-shot (QS) regimen being a commonly used RT schedule derived from prior palliative experiences in advanced pelvic malignancy [18,19]. This regimen comprises four 3.5- to 3.7-Gy fractions delivered twice daily over the course of two consecutive days [19–24]. This regimen is associated with favorable palliative response rates at the treated site and has been successfully delivered concurrently with cytotoxic chemotherapy (Table 3) [23,25]. Because of the nature of the hypofractionated regimen and its delivery in split-course cycles, acute toxicity is modest (Table 3).

**Table 3.** Response rates in published studies of quad-shot palliative radiotherapy for head and neck cancer.

Study	Dose, Gy/number of fractions	Response rate, %	Toxicity rate, %	Details	Ref.
Paris *et al.*	14.8/four	Complete: 28 Partial: 49	NA	One to three cycles delivered with 3–4 weeks between cycles.	[19]
Corry *et al.*	14/four	Complete: 6 Partial: 47	Mucositis G1: 33; G2: 11; G3: 0	One to three cycles delivered with 3–4 weeks between cycles.	[21]
Lok *et al.*	14.8/four	Pain: 66	Mucositis/dermatitis G3: 5	One to three cycles delivered with 3–4 weeks between cycles.	[20]
Finnegan *et al.*	14.8/four	Complete pain: 39 Partial pain: 22	Mucositis/dermatitis G2+: 26/17	One to three cycles delivered with 3–4 weeks between cycles.	[22]
Chen *et al.*	14.8/four	Palliative: 83	G3+: 9	One to three cycles delivered with 3–4 weeks between cycles.	[24]
Carrascosa *et al.*	14.8/four	Complete: 14 Partial: 71	Mucositis G3: 14	One to three cycles delivered with 3–4 weeks between cycles. Concurrent paclitaxel administered 1 h prior to the first fraction of each cycle.	[23]
Gamez *et al.*	14.8/four	Complete: 24 Partial: 62	Mucositis/xerostomia G2: 35	One to three cycles delivered with 3–4 weeks between cycles. Concurrent carboplatin or cetuximab administered 1 h prior to the first fraction of each cycle.	[25]

G: Grade; NA: Not applicable.

In prior studies, approximately 64% of patients were eligible to receive more than one QS cycle [20]. The equivalent dose in 2-Gy fractions (assuming α/β = 10 [tumor]) for a single cycle is 16.9 Gy, with 33.8 Gy for two cycles and 50.7 Gy for three cycles. The palliative response rates were reported to correlate with number of cycles delivered: 55% for one cycle, 44% for two cycles and 88% for three cycles (correlation coefficient: 0.29; p = 0.012). Despite the favorable rates of achieving palliation at the treated site, this regimen is insufficient for long-term disease control. Median duration of control reported in a phase II study was 5.7 months at the primary site and 5.2 months at nodal sites for those with complete or partial response and 1.8 months at the primary site and 9.5 months at nodal sites for those with stable disease [21]. Most patients will eventually experience local and/or regional progression that may result in significant morbidity or even mortality. Therefore, there is a foundation of evidence to support investigating the combination of QS RT and systemic therapies such as ICIs to prolong survival in patients with A/R/M HNSCC.

### Rationale for the combination of QS RT & immunotherapy

The mechanism of conventionally fractionated (1.8–2 Gy per fraction) RT is to induce clonogenic cell death in target cells by generating irreparable DNA damage. This is achieved indirectly through the generation of radical species such as reactive oxygen species and directly by inducing DNA damage [26]. Because T cells are sensitive to low doses of radiation, it is thought that conventionally fractionated RT over the course of several weeks may diminish the tumor immune response via local depletion of tumor-specific immune cells [27]. Hypofractionated RT, conversely, has been reported to modulate many components of the tumor microenvironment, including CD8+ T cells, Tregs, dendritic/antigen-presenting cells, expression of cytokines, IFN-γ and MHC [28].

Immune checkpoint inhibition has been studied in combination with RT in preclinical models with the goal of increasing ICI efficacy through RT-mediated escalation of tumor immunogenicity [29]. The combination of hypofractionated RT and anti-CTLA-4 antibodies or PD-1 pathway blockade appears to enhance the effects of RT on tumor cell kill [30–32]. At the same time, RT may resensitize tumors resistant to anti-PD-1 therapy, causing regression not only in the RT-targeted primary tumor but also in nonirradiated (non-RT-targeted) tumors [33]. It has been shown that tumor cells and other cells in the tumor microenvironment upregulate PD-L1 expression after RT, and the combination of ICI and hypofractionated RT may increase tumor kill by enhancing the efficacy of established T-cell-mediated tumor immunity [34].

The combination of ICI and RT was safe for patients with intermediate- or high-risk HNSCC in preliminary studies [25,26]. Early-phase studies have also demonstrated the safety of hypofractionated (3 Gy per fraction) regimens and stereotactic body RT, a technique that employs larger (8 Gy) fraction sizes [35,36]. These data support further clinical study of the efficacy of QS RT combined with ICI therapy. The central hypothesis is that a hypofractionated schedule of RT such as the QS scheme, utilized as single or repeated administrations, will be able to revert immune-resistant tumor phenotypes, increasing the tumor response rate to the combined treatment.

**Figure 1.**
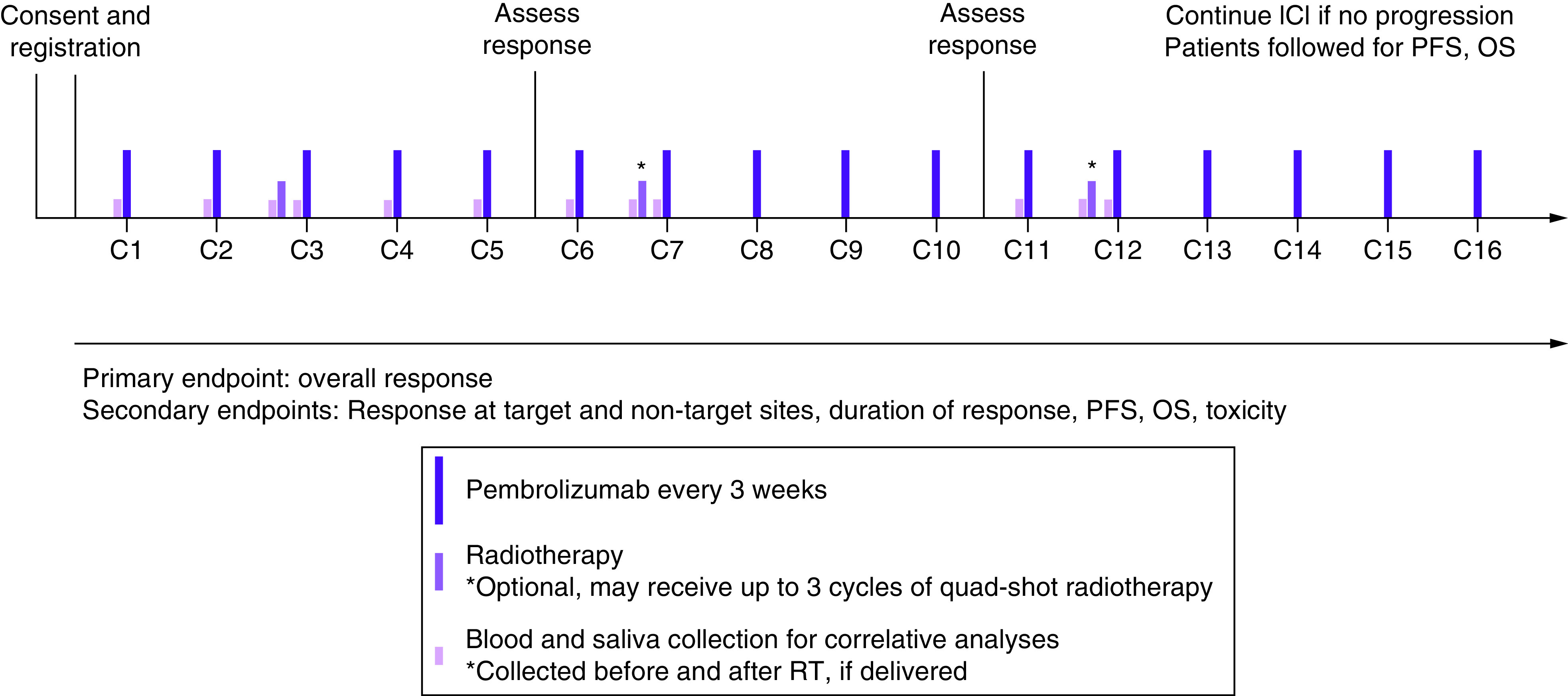
Protocol timeline for a pilot study of pembrolizumab immune checkpoint inhibition in combination with quad-shot radiotherapy for patients with advanced/recurrent head and neck cancer. ICI: Immune checkpoint inhibitor; OS: Overall survival; PFS: Progression-free survival; RT: Radiotherapy.

No clear clinical evidence exists to support the timing of ICI combined with RT. In a mouse model, a synergistic effect was described when RT was given in the days prior to ICI or on the same day [37]. Other preclinical evidence points to a benefit when the ICI is administered prior to RT [34,38]. The administration of ICI and RT on the same day is avoided out of concern for increased RT-related and immune-mediated adverse effects, although recent and ongoing clinical trials are testing the efficacy of conventionally fractionated RT concurrent with ICI (RTOG 3504: NCT02764593, HN-005: NCT03952585). Current standard of care for patients receiving palliative RT while on ICI or chemotherapy–ICI is to deliver RT between administrations of systemic therapy. The half-life of pembrolizumab is on the order of 14–27 days and reaches a steady state after approximately six cycles every 3 weeks [39]. We hypothesize that scheduling QS RT to occur after three initial cycles of pembrolizumab will provide sufficient PD-L1 blockade at the time of QS to prompt the tumor immunogenicity incited by RT (Figure 1). It has been suggested that RT doses in the range of 14–24 Gy in two to three fractions may be the most appropriate to combine with concurrent ICI [29], and the QS regimen falls close to this range (14.8 Gy in four fractions). Additionally, prior reports indicate that this regimen may be able to produce an abscopal effect in HNSCC patients with metastatic lesions treated with ICI [40].

## QS–immunotherapy clinical trial

We report the design and methods of a single-center study to investigate the efficacy and tolerability of a short course (or courses) of QS RT combined with the PD-1 inhibitor pembrolizumab. These standard-of-care treatments have the potential to act synergistically to improve response, and this study will investigate the combination of these two treatments in a novel sequence. The clinical hypothesis is that the delivery of one or more courses of QS RT in the proposed sequence with pembrolizumab is feasible and improves both response rates and duration of tumor response. This study will be conducted at the Wake Forest Baptist Comprehensive Cancer Center and is registered on ClinicalTrials.gov (NCT04454489).

### Study design

This is a single-arm, nonrandomized study to evaluate the feasibility, efficacy and tolerability of the combination of QS RT and pembrolizumab immunotherapy for patients with A/R/M HNSCC. Fifteen eligible patients will be enrolled and treated with standard of care pembrolizumab at a dose of 200 mg every 3 weeks until progression or treatment intolerance [41]. QS RT consisting of 14.8 Gy in four fractions delivered twice daily over two consecutive days will be delivered between cycles of pembrolizumab for up to three total courses per the study schedule presented in Figure 1. All patients will receive at least one cycle of QS RT before cycle four of pembrolizumab (between cycles two and three or three and four). Up to three QS treatments may be administered per protocol if deemed appropriate by the treating radiation oncologist: a second QS treatment prior to cycle eight of pembrolizumab (between cycles six and seven or seven and eight) and a third QS treatment prior to cycle 13 of pembrolizumab (between cycles 11 and 12 or 12 and 13). RT is not to be given on the same day as pembrolizumab infusion and is preferably given on days immediately preceding pembrolizumab administration. Though specific days are not mandated, it is strongly recommended that at least 1 day of the two consecutive days (including two of the four planned RT fractions per cycle) falls within 7 days before immunotherapy infusion. Palliative local (nonsystemic) interventions to other sites that are not QS RT target sites are allowed for the management of cancer symptoms.

Whenever tumor tissue is available, PD-L1 level will be tested using the Dako 22C3 assay [41], and results will be reported as CPS. HPV status will be determined by PCR or p16 immunohistochemistry, as an established surrogate marker, per standard of care when tissue is available. Next-generation sequencing is encouraged and will be done using blood or available tumor tissue whenever possible.

Patients will be removed from the treatment portion of the protocol if one of the following criteria applies: disease progression (not amenable to localized treatment), unacceptable adverse events, intercurrent illness that prevents further administration of treatment, patient decision to withdraw from the study or changes in patient condition rendering the patient ineligible for further treatment in the judgment of the treating oncologist. New patients will be enrolled to replace those who do not complete at least one QS treatment, those who do not undergo initial imaging response assessment and those who cannot provide scheduled correlative blood and saliva samples.

Saliva and blood will be collected for correlative studies. A comprehensive translational analysis will interrogate the molecular and cellular immune response to ICI alone and in combination with RT in order to generate a proposed immune-sensitive profile and to describe the mechanisms of immune sensitization with hypofractionated RT in immune unresponsive patients.

### Eligibility criteria

Eligibility and exclusion criteria are summarized in Table 4. Patients aged 18 years or older with A/R/M HNSCC and no curative localized treatment options and a target site in the head and neck region amenable to QS and who are eligible for palliative ICI (pembrolizumab) will be enrolled. Eastern Cooperative Oncology Group performance status must be 2 or less, and organ and marrow function must be within normal parameters. Patients will be excluded if they were treated with RT or any other anticancer agent within the 30 days prior to registration or with any prior PD-1 or PD-L1 inhibitor therapy or have participated in a clinical trial within 3 months, have active contraindications to RT or have any relative or absolute contraindications to ICIs.

**Table 4.** Inclusion and exclusion criteria for the immunotherapy combined with quad-shot radiotherapy study protocol.

Inclusion criteria
• A/R/M HNSCC as defined by clinical or pathological diagnosis of any of the following: ○ Locally advanced HNSCC not suitable for curative local treatment ○ Locally recurrent HNSCC not suitable for curative local treatment within or outside previously irradiated tissue ○ Metastatic HNSCC • Target site in the head and neck region amenable to QS palliative RT, for which palliative RT is recommended, as determined by the treating radiation oncologist • Age 18 years or older at time of registration • ECOG performance status of 0–2 • Women of child-bearing age, and men must agree to use adequate contraception (hormonal or barrier method of birth control, abstinence) prior to study entry and for the duration of study participation. Should a woman become pregnant or suspect she is pregnant while participating in this study, she should inform her treating physician immediately • Ability to understand and willingness to sign an IRB-approved informed consent document (either directly or via a legally authorized representative). • Willingness to provide blood and saliva samples for exploratory research purposes • Organ and marrow function as defined by the following: ○ ANC ≥1.5 × 10^9^/l ○ Platelet count ≥100 × 10^9^/l ○ Hemoglobin ≥9.0 g/dl ○ Serum bilirubin ≤1.5 × institutional ULN ○ AST and ALT ≤2.5 × institutional ULN ○ Serum creatinine clearance >40 ml/min by the Cockcroft–Gault formula or by 24-h urine collection for determination of creatinine clearance

**Exclusion criteria**
• RT to the planned QS RT target region within 30 days of registration • Prior RT to the head and neck that precludes safe delivery of study RT as determined by the treating radiation oncologist. • Active medical conditions that are contraindications to study RT (e.g., scleroderma) as determined by the treating radiation oncologist • Pregnant or lactating women are excluded from this study because RT is contraindicated in pregnancy and because there is an unknown but potential risk of adverse events in nursing infants secondary to treatment of the mother with immunotherapy • Participation in another clinical study with an investigational product during the last 3 months • Any previous treatment with a PD-1 or PD-L1 inhibitor. • Any anticancer therapy (chemotherapy, immunotherapy, endocrine therapy, targeted therapy, biologic therapy, tumor embolization, monoclonal antibodies, other investigational agent) within the last 30 days. Note: this excludes palliative RT to the nontarget site • Mean QTc ≥470 ms, except for patients with pacemaker who have a paced ventricular rhythm • Current or prior use of immunosuppressive medication within 30 days, with the exception of intranasal and inhaled corticosteroids; brief, nonsustained corticosteroid treatment for incidental problems such as allergies (at the discretion of the treating physician); or sustained systemic corticosteroid treatment at physiological doses, not to exceed 10 mg/day of prednisone or an equivalent corticosteroid • Any unresolved toxicity (CTCAE grade >2) from previous anticancer therapy. Subjects with irreversible toxicity that is not reasonably expected to be exacerbated by the investigational product (e.g., hearing loss, peripheral neuropathy) may be included • Any prior grade ≥3 irAE while receiving any previous immunotherapy agent or any unresolved grade >1 irAE • Active or prior documented autoimmune disease within the past 2 years. Note: subjects with vitiligo, Graves' disease or psoriasis not requiring systemic treatment (within the past 2 years) are not excluded • Active or prior documented inflammatory bowel disease (e.g., Crohn's disease, ulcerative colitis) • History of primary immunodeficiency. • History of allogeneic organ transplant • History of hypersensitivity to any excipient in pembrolizumab • History of pneumonitis or interstitial lung disease • Subjects with uncontrolled seizures. • Uncontrolled intercurrent illness, including but not limited to ongoing or active infection, symptomatic congestive heart failure, uncontrolled hypertension, unstable angina pectoris, uncontrolled cardiac arrhythmia, active peptic ulcer disease or gastritis, active bleeding diathesis, evidence of acute or chronic hepatitis B or hepatitis C or HIV or psychiatric illness/social situations that would limit compliance with study requirements or compromise the ability of the subject to give written informed consent • Known history of active tuberculosis • Receipt of live attenuated vaccination within 30 days prior to study entry or within 30 days of receiving pembrolizumab • Any condition that, in the opinion of the investigator, would interfere with evaluation of study treatment or interpretation of patient safety or study results.

ALT: Alanine transaminase; ANC: Absolute neutrophil count; A/R/M: Advanced, recurrent or metastatic; AST: Aspartate aminotransferase; CTCAE: Common Terminology Criteria for Adverse Events; ECOG: Eastern Cooperative Oncology Group; HNSCC: Head and neck squamous-cell carcinoma; irAE: Immune-related adverse event; IRB: Institutional review board; QS: Quad-shot; QTc: QT interval corrected for heart rate; RT: Radiotherapy; ULN: Upper limit of normal.

### Study objectives

The primary objective is to determine the overall response rate for pembrolizumab given with QS RT. Secondary objectives are to determine the following: response rate at the target and nontarget lesions, durability of response at the target lesion, PFS, OS and tolerability of the combination of QS RT and pembrolizumab. The exploratory objectives are to evaluate the effect of QS on pembrolizumab-mediated immune activation and to determine possible mechanisms of any observed immunosensitizing effects of the proposed ICI plus QS RT treatment regimen.

### Study end points & procedures

The primary outcome measure is the overall treatment response rate according to Response Evaluation Criteria in Solid Tumors 1.1. Follow-up imaging with CT, MRI or PET/CT will be obtained every five to six immunotherapy treatments. For the purposes of tumor measurements, follow-up imaging will be performed using the same modality (CT or MRI) used for baseline imaging measurements. PET/CT may be utilized for follow-up imaging at the clinician's discretion. Response in the target lesion(s) and in other, nontarget head and neck lesions will be assessed and documented during the course of the study by a fellowship-trained neuroradiologist with subspecialty expertise in head and neck imaging. The maximum response documented in the target lesion(s) will address the primary objective. Duration-of-response at the target lesion(s) will be defined as the duration of time from when the measurement criteria are met for complete or partial response (whichever is recorded first) to the first date of documented recurrent or progressive disease. PFS will be defined as the duration of time from registration to the time of progression, death or date of last contact; patients lost to follow-up will be censored. OS will be defined as the duration of time from registration to the date of death or date of last contact; patients lost to follow-up will be censored. The study treatment ends for all patients with the last administration of immunotherapy. Patients will be followed after the conclusion of treatment for PFS and OS outcomes. Subsequent visits can be accomplished in person or over the phone, and clinical and imaging data will be collected from medical records as available. Treatment toxicities will be monitored and documented during the treatment and for 30 days from the end of immunotherapy. Toxicities will be graded according to Common Terminology Criteria for Adverse Events (CTCAE) version 5.0. Patient-reported outcomes will be obtained at each time point using a targeted checklist of head and neck cancer-specific Patient-Reported Outcome–CTCAE version 1.0 items.

Saliva and blood will be collected before each ICI treatment for the first five cycles and before RT on the first day of each QS treatment. For patients treated with subsequent QS cycles (i.e., second or third QS), samples will be collected before the ICI cycles that are administered before and after each additional QS. Levels of immunoregulatory miRNAs will be quantified in plasma using PCR-based techniques. Immune miRNA analysis will be utilized to create an immune response curve in order to select the best-response sample for in-depth immune response investigation, employing targeted analysis with single-cell RNA sequencing and complementary mass spectrometry-based omics (proteomics, metabolomics and lipidomics) on peripheral blood mononuclear cells and plasma. We will utilize an integrated data processing pipeline to align and interpret multiomics profiles to identify molecular drivers of response to ICI combined with QS RT. Expression of the top five candidate biomarkers identified, as determined by fold change and statistical significance, will be secondarily validated by western blot or mass spectrometry-based targeted analysis. Oral microbiota profiling will be performed using 16S rRNA gene sequencing to characterize the effect of ICI and the combination with QS RT on bacterial diversity and composition in saliva.

### Treatment

#### Quad-shot RT

The gross tumor volume will be defined as the primary and/or nodal disease to be targeted. The clinical target volume will be defined as areas at risk of subclinical disease around the gross tumor volume that may be delineated at the discretion of the treating radiation oncologist. The planning target volume will be a 0.3- to 1-cm isotropic expansion from the gross tumor volume or clinical target volume (if present). Each cycle of QS RT will comprise 14.8 Gy in four fractions (3.7 Gy per fraction) delivered twice daily (at least 6 h apart) over two consecutive days before the administration of pembrolizumab. All patients will receive at least one cycle of QS RT; up to three QS cycles may be administered per protocol if deemed appropriate by the treating radiation oncologist. The first administration of QS RT will occur before cycle four (between cycles two and three or three and four) of pembrolizumab. If deemed appropriate by the treating radiation oncologist, a second QS may occur prior to pembrolizumab cycle eight (between cycles six and seven or seven and eight) and a third QS may occur prior to pembrolizumab cycle 13 (between cycles 11 and 12 or 12 and 13). Therefore, the total RT dose over the course of the study will depend on the number of QS RT treatments delivered: 14.8 Gy in four fractions for those who complete one QS RT cycle, 29.6 Gy in eight fractions for those who complete two QS RT cycles or 44.4 Gy in 12 fractions for those who complete three QS RT cycles. Upon planning the initial cycle of QS RT, a composite plan will be generated to assess the total dose to the target and organs at risk. Repeat CT simulation may be performed in order to plan subsequent cycles. QS RT will not be given on the same day as pembrolizumab infusion, and it is recommended to occur no more than 7 days prior to the next pembrolizumab infusion.

### Statistics

#### Planned sample size

For this single-arm pilot study, the planned sample size is 15 patients. With a sample size of 15 patients, the maximum width of a Clopper–Pearson exact 95% CI is 0.52. Since the interval may not be symmetric, this corresponds to approximately ± 0.26, if the response rate is as high as seven of 15. If the response rate is lower than this, the width of the CI will be less. With this sample size, we will be able to determine whether the response rate in this treatment is sufficient to warrant future examination. Based on the preliminary data presented in Table 1, the reported response rates from previous studies range between 13 and 36%. Thus, we would anticipate that the response rate of the combined QS RT and pembrolizumab regimen used in this study will need to be at least this high to warrant future studies.

#### Planned study period

According to internal metrics, our institution saw an average of 2.6 patients meeting these inclusion criteria per month over the 3 years prior to study design, and 3.5 arrivals per month were expected over the following year. Considering ineligibility and patient declination, we expect to accrue 15 patients over the course of 2 years.

#### Analysis plan

The primary analytic objective is to measure the overall response rate according to Response Evaluation Criteria in Solid Tumors 1.1. We also aim to determine the percentage of patients with either a complete or partial response as well as the corresponding Clopper–Pearson exact 95% CI around this measure. These results will provide useful information concerning the potential efficacy of this treatment for planning future studies.

There are six secondary outcomes of interest for this pilot study: 1) response rate for the target lesion(s), 2) response rate for the nontarget lesion(s), 3) duration-of-response for the target lesion(s), 4) PFS, 5) OS and 6) tolerability assessed using Patient-Reported Outcome–CTCAE. The analytical approach for each measure is as follows: 1) for the response rate measures, we will estimate the percentage of responders (complete or partial response) and corresponding Clopper–Pearson exact 95% CI; 2) for the duration-of-response among responders, we will estimate the mean and median response duration, with the corresponding 95% CI for the mean and interquartile range for the median; 3) for time-to-event measures (PFS and OS), we will estimate Kaplan–Meier survival curves and median time-to-event times as well as percent PFS and OS at 6 months and 1 year post-treatment; 4) for tolerability, we will estimate the percentage of patients with different adverse events assessed using the Patient-Reported Outcome–CTCAE and corresponding Clopper–Pearson exact 95% CI.

Additional exploratory subgroup analyses are limited by the small sample size but will be considered if adequate sample size is achieved in each subgroup. A main consideration for exploratory analysis will be to compare response rates and other oncological outcomes (duration-of-response, PFS, OS) between patients with HPV-associated oropharyngeal squamous-cell carcinoma and those with non-HPV-associated oropharyngeal cancer/nonoropharyngeal HNSCC.

## Conclusion

In order to improve therapeutic outcomes for patients with A/R/M HNSCC, we aim to assess response to, survival associated with and toxicity of a novel combination of pembrolizumab and QS RT. Extensive laboratory correlative analyses are planned to elucidate the immune-related impact of this RT regimen combined with ICI. Additionally, the data provided by this clinical trial may provide evidence supporting future confirmatory clinical trials investigating this paradigm.

**Executive summary**

**Advanced/recurrent head & neck squamous-cell carcinoma**

- Of patients with head and neck squamous-cell carcinoma, up to 20–30% develop metastatic disease and up to 40–60% experience locoregionally recurrent disease.
- Systemic therapy alone is associated with limited response rate and survival.
- Quad-shot (QS) radiotherapy (RT) is a low-dose palliative regimen that provides symptomatic relief and local control.

**QS RT in combination with immunotherapy**

- The tumor microenvironment is characterized by changes that shield cancer cells from immune recognition and targeting.
- Palliative hypofractionated RT induces changes in the immunoregulatory microenvironment, such as increased tumor immunogenicity, induction of MHCs, alteration of cytokine expression and changes in T-cell and antigen-presenting cell function.
- Hypofractionated RT (e.g., QS) is considered to optimally synergize with immune checkpoint inhibition.

**QS-immunotherapy trial**

- In this single-center study, 15 patients will be treated with pembrolizumab at a dose of 200 mg every 3 weeks.
- QS RT (14.8 Gy in four twice-daily fractions) will be delivered at least once prior to cycle four of pembrolizumab and up to three times (before cycles eight and 13).
- Overall response, target and nontarget lesion response, durability of response, progression-free survival, overall survival and tolerability will be measured.
- Integrative multiomics, including proteomics, metabolomics, immunoregulatory miRNA expression, oral microbiome analysis and single-cell RNA sequencing, will be performed on blood and saliva samples collected before and during treatment to evaluate the effect of QS RT on the immune response to pembrolizumab.

**Conclusion**

- This study of a novel combination of QS RT and pembrolizumab is the first of its kind.
- Preliminary results will inform future clinical trials, and planned correlative analyses will improve understanding of the mechanisms of pembrolizumab combined with RT in advanced/recurrent head and neck squamous-cell carcinoma.
